# Epitaxial synthesis of Ni–MoS_2_/Ti_3_C_2_T_*x*_ MXene heterostructures for hydrodesulfurization†

**DOI:** 10.1039/d0ra01158d

**Published:** 2020-03-26

**Authors:** Mari Vinoba, R. Navvamani, Hanadi Al-Sheeha

**Affiliations:** Petroleum Research Center, Kuwait Institute for Scientific Research Kuwait vmari@kisr.edu.kw vinoba76@gmail.com

## Abstract

Hierarchical structures of 2D layered Ti_3_C_2_T_*x*_ MXene hold potential for a range of applications. In this study, catalysts comprising few-layered MoS_2_ with Ti_3_C_2_T_*x*_ have been formulated for hydrodesulfurization (HDS). The support Ti_3_C_2_T_*x*_ was derived from MAX phases (Ti_3_AlC_2_) *via* a liquid-phase exfoliation process, while MoS_2_ was obtained from synthesized aqueous ammonium tetrathiomolybdate (ATM). Furthermore, a series of catalysts with different architectures was synthesized by confinement of ATM and/or the promoter Ni in Ti_3_C_2_T_*x*_ at different mole ratios, through a thermal conversion process. The synthesized MoS_2_/Ti_3_C_2_T_*x*_ and Ni–MoS_2_/Ti_3_C_2_T_*x*_ catalysts were characterized using X-ray diffraction (XRD), Brunauer–Emmett–Teller (BET), scanning electron microscopy coupled with energy dispersive X-ray spectroscopy (SEM-EDS), high-resolution transmission electron microscopy (HRTEM), and temperature-programmed reduction (TPR) measurements. The number of MoS_2_ layers formed on the Ti_3_C_2_T_*x*_ support was calculated using Raman spectroscopy. The heterostructured few-layered MoS_2_/Ti_3_C_2_T_*x*_ catalysts were applied in sulfur removal efficiency experiments involving thiophene. The active MoS_2_ sites confined by the Ti_3_C_2_T_*x*_ enhanced hydrogen activation by proton saturation, and the electron charge stabilized the sulfur atom to facilitate hydrogenation reactions, leading to predominant formation of C_4_ hydrocarbons. The Ni–MoS_2_/Ti_3_C_2_T_*x*_ showed the best activity at a promoter molar ratio of 0.3 when compared to the other catalysts. In particular, it is evident from the results that ATM and Ti_3_C_2_T_*x*_ are potential materials for the *in situ* fabrication of hierarchical few-layered MoS_2_/Ti_3_C_2_T_*x*_ catalysts for enhancing hydrodesulfurization activity in clean fuel production.

## Abstract 2

A facile and efficient method is adopted to intercalate promoter and few-layered MoS_2_ in Ti_3_C_2_T_*x*_ MXene for high hydrodesulfurization activity *via* an *in situ* thermal conversion process.

## Graphical abstract

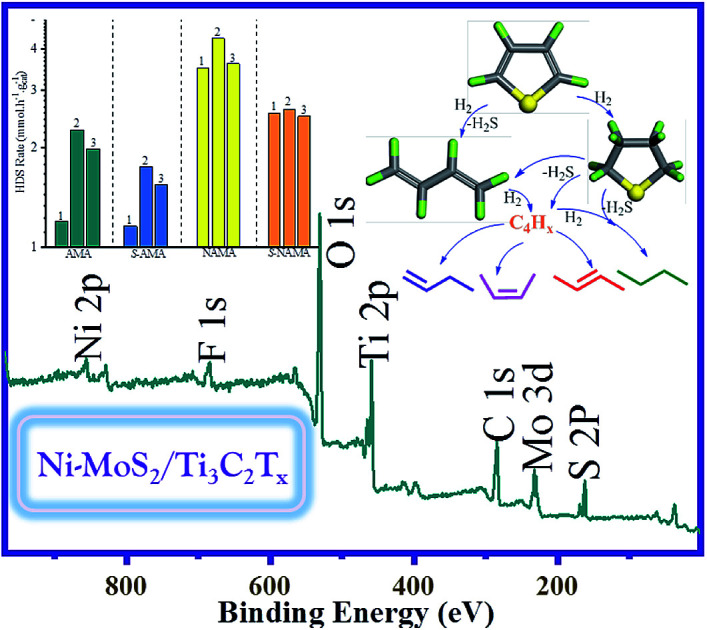

## Introduction

The current socio-economic climate demands control over production costs, innovative technologies, zero pollution to life-giving sources, and “no waste” management in petroleum refineries. Many factors affect the goals of petroleum refineries, and one of these is the effect of sulfur emitted from automotive fuels to the environment; thus environmental protection agencies have imposed stringent regulations on sulfur levels in transportation fuels.^1,2^ Over recent years, many conventional supported and unsupported NiMo and CoMo-sulfide catalysts have been employed in the hydrodesulfurization (HDS) reaction.^3,4^ The supported catalysts show more stability, offer enhanced dispersion of active metal sites on the surface, and are more durable than unsupported catalysts. The prominent activity of hydrotreating catalysts depends on the potential of the active sites, and the structure and textural characteristics of the supports.^5–7^ Generally, alumina is used as a support as it is economical and possesses unique physicochemical properties. However, the conventional sulfidation of Mo-oxide on alumina with H_2_S treatment occurs at a high temperature, which may lead to the formation of a mixture of Mo-sulfides/oxides, and the inhibition of HDS activity due to strong metal–alumina interactions.^8–10^ Furthermore, promoted hydrotreating catalysts show enhanced HDS activity due to promoter atoms which are dispersed on the MoS_2_ edges of the square pyramidal structure in the same plane as the Mo atoms.^11,12^ It is reported that the morphology of promoter–Mo–sulfur catalysts with supports is characterized either by a highly dispersed single-layer structure that strongly interacts with the support and has low sulfur coordination with Mo and the promoter (Type-I) or by a less dispersed few-layered structure that weakly interacts with the support due to complete sulfidation and has higher sulfur coordination to Mo and the promoter (Type-II). Due to the structural properties, it has been reported in previous studies that the Type-II structure reveals higher HDS activity than the Type-I structure.^13,14^ Notably, a few reports have described the use of ammonium thiometallates as precursors for the preparation of active metal-sulfide HDS catalysts due to their unique electronic structures and chemical reactivity.^15,16^ Currently, the thermal conversion of ammonium thiomolybdate at a low temperature is used to produce uniformly active MoS_2_ dispersed on supported HDS catalysts; this could provide a more economical method than conventional sulfidation of molybdenum oxides. Moreover, the thiomolybdate precursor contains sulfur bonded to Mo in a tetrahedral coordination, which undergoes a topotactic reaction during the thermal conversion; as a result, the *c*-axis of sulfide is retained in the precursor.^17^

In recent years, metal carbides have played a significant role in the formulation of new generation catalysts with novel support systems that can overcome the inhibition of HDS efficiency due to strong metal–support interactions. This hybrid system composed of a support and active metals enhances the removal of sulfur through the breaking of the ring S–C bonds in thiophene derivatives.^18,19^ In this regard, transition metal carbides and nitrides that possess significantly altered physical and chemical properties have been recognized as encouraging support materials for crude oil hydroprocessing due to their excellent performance and stability in HDS processes.^7,20^ Transition metal carbides have attracted attention as prospective catalysts for use in the HDS process as they exhibit hydrogenating properties similar to those of noble metals,^21^ and at the same time, they are sulfur-tolerant.^22^ Currently, however, researchers are giving more attention to the new family of two-dimensional (2D) transition metal carbides and nitrides (MXenes) (where M refers to early transition metals (groups 13 and 14) and X corresponds to carbon and/or nitrogen) because of their unique structure, mechanical, optical and electronic properties, large specific surface areas, surface hydrophilicity, and tunable compositions.^23^ MXenes (M_*n*+1_X_*n*_T_*x*_) (where T_*x*_ denotes surface terminated functional groups (–F, –OH, and –O)) are derived from MAX phases (M_*n*+1_AX_*n*_) (where A stands for mostly IIIA or IVA group elements) by selective etching of the A layer. The ordered MXenes comprise few atoms that have *n* layers of sandwiched Ti–C–Ti sheets. MXene-based hybrid materials play a vital role in a wide range of applications such as energy storage,^24^ hydrogen storage,^25^ catalysis,^26^ sensing,^27^ water purification,^28^ and gas separation^29^ due to the attached surface anion functional groups, which provide hydrophilic surfaces and enhance the dispersion of the catalysts.

Based on the above, the MXene Ti_3_C_2_T_*x*_ was derived by etching the Al-layer from a MAX compound Ti_3_AlC_2_. A few-layered MoS_2_ catalyst was developed by combining the active metal and promoter on the MXene support (Ni–Mo–S/Ti_3_C_2_T_*x*_). It was investigated for thiophene HDS at a low hydrogen pressure and reaction temperature. Synthesized ammonium thiomolybdate (ATM) was used as a precursor for the preparation of the MoS_2_ catalyst. The few-layered MoS_2_ was derived by controlled calcination in an inert medium in a rotary furnace.

## Experimental

### Preparation of Ti_3_C_2_T_*x*_ MXene and ATM solution

Ti_3_AlC_2_ powder was dispersed slowly in 30% HF solution in a ratio of 1 : 10 (Ti_3_AlC_2_ : HF) (**Caution**: exothermic reaction) with continuous stirring at room temperature for 48 h. This exfoliated the MXene layers by etching the aluminum. The de-aluminated product MXene (Ti_3_C_2_T_*x*_) was washed in water and centrifuged several times until the pH reached neutral. Then it was dried at 60 °C for 12 h.^30^ A stock solution of ATM was prepared by dissolving ammonium heptamolybdate (15.8 mmol) in a mixture of ammonium hydroxide and water.^31^ The mixture was purged with 10% hydrogen sulfide (H_2_S/Ar) at 100 mL min^−1^ for sulfidation at room temperature for 2 h. The temperature was gradually increased to 60 °C with continuous H_2_S purging until a dark red stock solution was obtained, and this was used as a precursor for MoS_2_.

### Formulation of the MoS_2_/Ti_3_C_2_T_*x*_ MXene catalysts

The incipient wetness impregnation technique was used to prepare two sets of Ti_3_C_2_T_*x*_ supported target catalysts. One set (termed AMA) was prepared with active MoS_2_ on Ti_3_C_2_T_*x*_, and the other (termed NAMA) was prepared using a Ni promoter with MoS_2_ on Ti_3_C_2_T_*x*_. A series of AMA catalysts were formulated at different ratios of Mo (6, 9, and 12 wt%) by dispersion of the ATM stock solution in the Ti_3_C_2_T_*x*_ support. These materials were dried and calcined in the rotary furnace under N_2_ and are denoted as AMA-1, AMA-2, and AMA-3, respectively. The NAMA catalysts were prepared by a stepwise impregnation method. First, ATM solutions (6, 9, and 12 wt% Mo) were impregnated on MXene, as above, and then the samples were impregnated with nickel nitrate. The Ni/(Ni + Mo) ratios for the samples with 6, 9 and 12 wt% Mo were maintained at 0.39, 0.30, and 0.24, respectively. Subsequently, the materials were calcined in an inert medium to give the NAMA catalysts denoted as NAMA-1, NAMA-2, and NAMA-3, respectively. The above-synthesized series of AMA and NAMA catalysts were next treated with 10% H_2_S (balanced H_2_) in a reactor at 375 °C for 4 h, and then the temperature was reduced to 350 °C for the thiophene HDS reactions; the corresponding catalysts are denoted as S-AMA and S-NAMA, respectively. All the synthesized materials were characterized and investigated for thiophene HDS activity.

### Catalytic properties of the MXene-based catalysts

The HDS activity of each synthesized catalyst was assessed in a fixed glass reactor associated with a thiophene saturator system. Hydrogen was bubbled through thiophene to attain an equilibrium between liquid thiophene and its vapor, and then the produced vent gas mixture (H_2_ + thiophene) was used as a feed.^32^ The thiophene concentration in the reactor inlet was quantified from its vapor pressure using the Antoine eqn (1); the molar fraction of thiophene + hydrogen is given by eqn (2).

1 ln *P*^sat^ (mm Hg) = *A* − *B*/(*C* + *T*_sat_)

2 *X*_thiophene_ = *P*^sat^/*P*

where *P* is the total pressure (mm Hg), *P*^sat^ is the vapor pressure (mm Hg), and *T*_sat_ is the saturator temperature (K) of thiophene. *X*_thiophene_ is a molar fraction. The calculated values were *P*^sat^ = 28.47 mm Hg (3.80 kPa), and *X*_thiophene_ = 0.0375. The Antoine coefficients *A* = 16.0243, *B* = 2869.07, and *C* = −58.8 were valid in the temperature range from 260 to 380 K.^33^ From the conditions adopted, it was calculated that the inlet gas mixture consisted of about 3.75 mol% thiophene in H_2_ when the saturator temperature was set at 5 °C.

The desired amount of catalyst was placed in the glass reactor and purged with hydrogen-containing thiophene vapor at 50 mL min^−1^ at 350 °C. The reactor outlet was connected to an online gas chromatograph (GC) for the quantification of C_1_–C_4_ hydrocarbon products and unreacted thiophene. The GC was calibrated with Agilent refinery gas mixture P/N 5190-0519.^31^ The data were recorded at periodic intervals to evaluate the conversion rate and product selectivity. The molar flow of thiophene into the reactor (3) and HDS conversion rate (4) were calculated as follows:

3 *F*_thiophene_ = *P*^sat^ × *V*/*RT*_sat_

4 Conversion rate (*ν*) = *F*_thiophene_ × *α*/*m*

where *F*_thiophene_ is the molar flow of thiophene (mol h^−1^), *ν* is the conversion rate (mol h^−1^ g^−1^), and *V*, *R*, *α* and *m* are the inlet total flow (L h^−1^), gas constant (0.082057 L atm mol^−1^ K^−1^), conversion fraction, and mass of catalyst (g), respectively.

## Results and discussion

### Characterization of the catalysts

The XRD patterns of the pristine MAX phase (Ti_3_AlC_2_), and MXene (Ti_3_C_2_T_*x*_) are shown in Fig. S1.† Ti_3_AlC_2_ displayed characteristic intense peaks at 2*θ* = 9.54°, 19.17°, 33.99°, 38.99° and 40.89° corresponding to the planes (002), (004), (101), (104) and (105), respectively. All the 2*θ* values agreed with those in JCPDS no. 52-0875, confirming the purity of Ti_3_AlC_2_.^34,35^ In the MXene pattern, the most intense peak of Ti_3_AlC_2_ located at 38.99° (104) was completely absent due to the HF-etching process that had removed the entire atomic aluminum layer. Also, the Al-layer exfoliated MXene exhibited shifts in two peaks toward lower angles, from 9.54° and 19.17° to 9.10° (002) and 18.50° (004). At the same time, two new peaks emerged at 2*θ* = 27.94° (008), and 60.78° (110),^25,36^ which confirmed the transformation of Ti_3_AlC_2_ to Ti_3_C_2_T_*x*_ MXene.

**Fig. 1:**
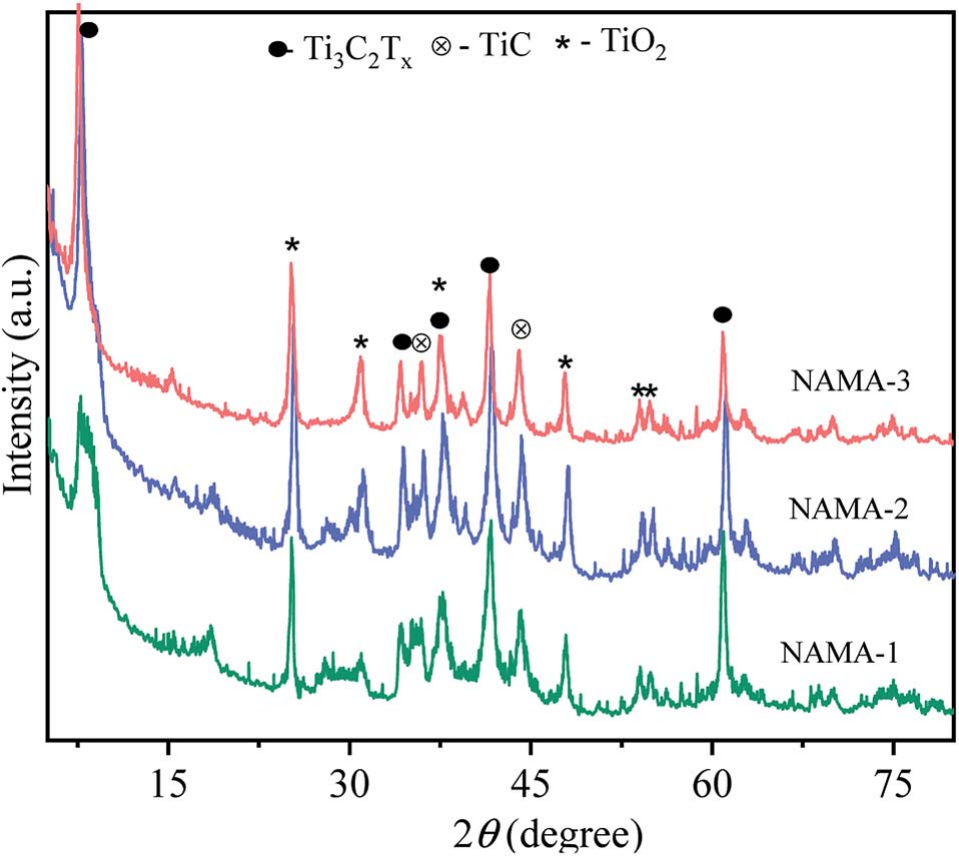
XRD diffraction patterns of the NAMA (NiMoS_2_/Ti_3_C_2_T_*x*_) catalysts.

The XRD patterns of the AMA (Fig. S2†) and NAMA (Fig. 1) catalysts revealed no individual MoS_2_ peaks (JCPDS 03-065-0160) due to the formation of nanocrystalline MoS_2_ on Ti_3_C_2_T_*x*_.^37^ Furthermore, the (002) peaks of AMA-1, AMA-2, AMA-3, NAMA-1, NAMA-2, and NAMA-3 catalysts were downshifted in comparison to that of Ti_3_C_2_T_*x*_ (9.10°) to ∼7.4°–8.3°. These shifts towards lower 2*θ* values confirmed the enlarged *d*-spacing of Ti_3_C_2_T_*x*_ due to the effective intercalation of MoS_2_ between the Ti_3_C_2_T_*x*_ layers in the prepared catalysts. In addition, the diffraction peaks of Ti_3_C_2_T_*x*_ were retained at their original 2*θ* positions of 37.6° and 60.78° (002); this indicated the persistence of Ti_3_C_2_T_*x*_ in the AMA and NAMA catalysts.^38^ The XRD patterns of the NAMA catalysts exhibited new peaks corresponding to TiC and TiO_2_ which could be attributed to the minor conversion of Ti_3_C_2_T_*x*_ to TiC and TiO_2_ in the presence of the promoter salt during the calcination, but these impurities were relatively very low in the AMA catalysts.

**Fig. 2:**
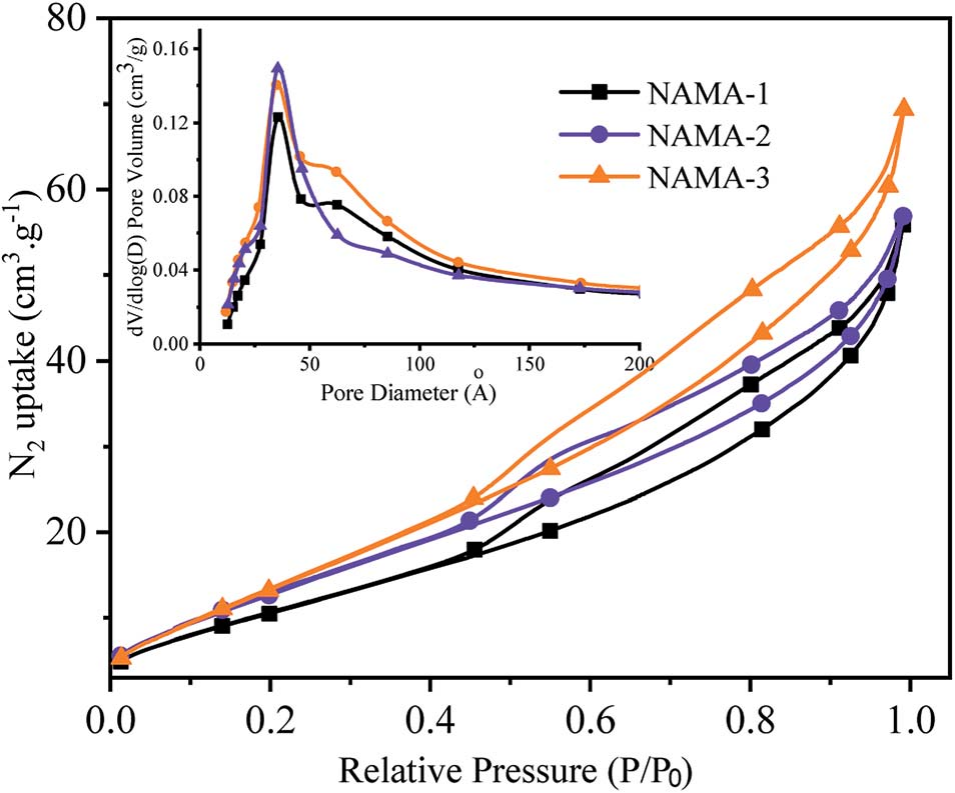
BET hysteresis loops and pore size profiles of the NAMA catalysts.

The N_2_ adsorption–desorption isotherms of the AMA (Fig. S3†) and NAMA (Fig. 2) catalysts show type H3 hysteresis. The specific surface areas of Ti_3_AlC_2_ and Ti_3_C_2_T_*x*_ calculated using the Brunauer–Emmett–Teller (BET) method were found to be 4.2 and 13.5 m^2^ g^−1^, respectively. All of the prepared catalysts showed increasing specific surface areas when the Mo loading was increased from 6 to 12%. For example, the BET surface areas of AMA-1, AMA-2, AMA-3, were 34.5, 42.7, and 47.3 m^2^ g^−1^, respectively, and the same trend was observed for NAMA-1, NAMA-2, and NAMA-3 which had surface areas of 45.1, 55.8 and 62.8 m^2^ g^−1^, respectively. The higher surface areas of the catalysts compared to the support (Ti_3_C_2_T_*x*_) were most likely due to the intercalation of MoS_2_ between the Ti_3_C_2_T_*x*_ interlayers and its anchoring on the surfaces along with the partial delamination of the Ti_3_C_2_T_*x*_ layers. In addition, the NAMA catalysts had higher surface areas than the AMA type catalysts due to the improved crystallinity of the nickel promoter as well as the MoS_2_ between the Ti_3_C_2_T_*x*_ interlayers. The pore size distributions indicate that the pores of the AMA and NAMA catalysts were in the mesoporous range (20–500 Å), and results show a decreasing trend from 50 to 40 Å with an increase in Mo loading from 6 to 12 wt%. Thus, the better textural properties of the AMA and NAMA catalysts should be beneficial for enhancing catalyst contact area, and facilitating the access of the reactant to the active MoS_2_ sites on the catalytic surfaces.

**Fig. 3:**
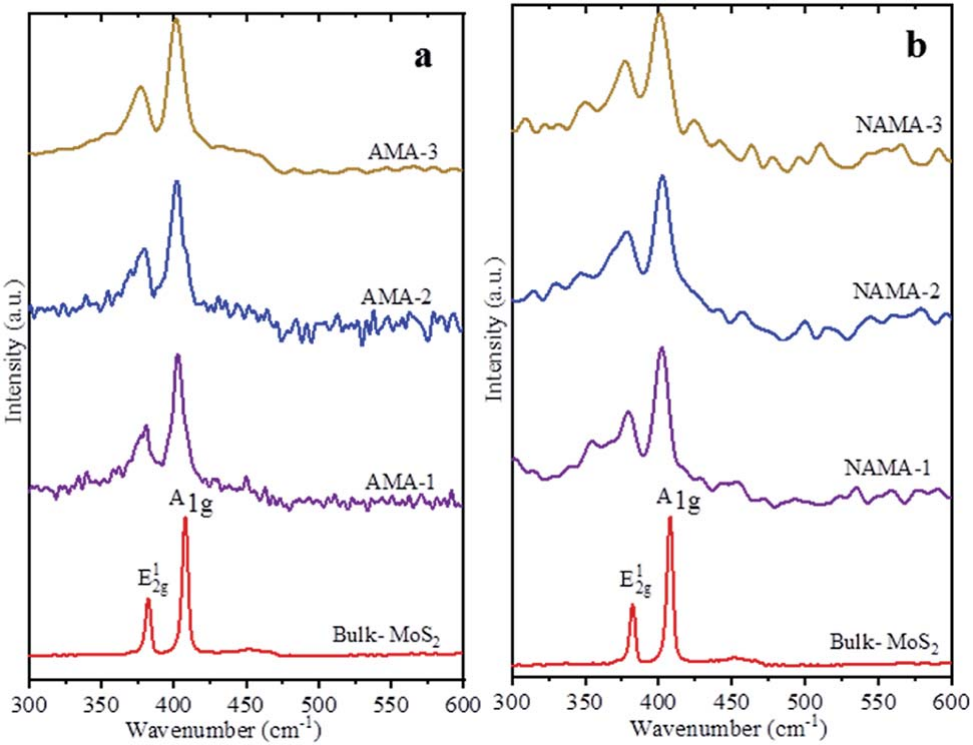
Raman spectra of the (a) AMA and (b) NAMA catalysts compared with the spectrum of bulk MoS_2_.

Raman spectra of the few-layered MoS_2_/MXene catalysts exhibited two characteristic MoS_2_ vibration modes, as shown in Fig. 3. The frequency displacement between the in-plane and out-of-plane Mo–S phonon modes, corresponding to the E^1^_2g_ vibration, softened (red-shifted), while the A_1g_ vibration stiffened (blue-shifted) in the catalysts.^38,39^ These two modes are thickness-dependent and indicate the number of layers.^40^ Typically, the difference in vibration modes of monolayer, few-layer, and bulk crystal MoS_2_ are 18, 19–24, and >25 cm^−1^, respectively. The few-layered MoS_2_ catalysts (AMA and NAMA) were successfully derived from the thermal conversion of ATM, and displayed active modes at ∼400 cm^−1^ (A_1g_) and ∼378 cm^−1^ (E^1^_2g_); the asymmetric stretching peak at 477 cm^−1^, and the symmetric vibration at 456 cm^−1^ of the Mo=S bond in tetrahedral [MoS_4_]^2−^ (ref. 31) were not detected. The frequency differences between the vibration modes for AMA-1, AMA-2 and AMA-3 were 22.0, 22.5, and 22.9 cm^−1^, respectively, while NANA-1, NAMA-2 and NAMA-3 had differences of 22.2, 22.5, and 23.1 cm^−1^, respectively. In contrast, the frequency difference between the E^1^_2g_ and A_1g_ modes for bulk MoS_2_ was 25.5 cm^−1^. Thus, the Raman spectroscopy results for the catalysts illustrated differences between the two vibration modes of ∼22.5 cm^−1^, which indicated the successful formation of few-layered MoS_2_ on MXene.

**Fig. 4:**
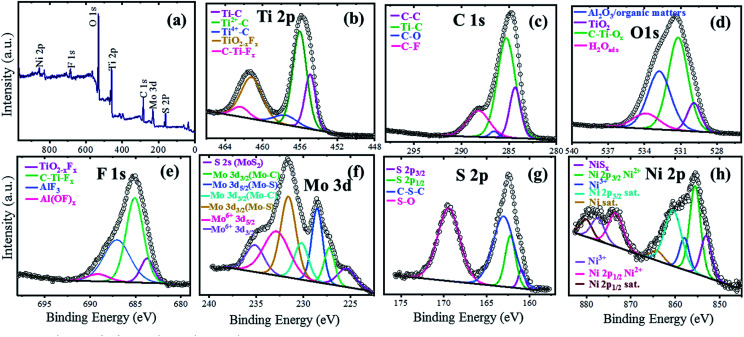
XPS spectra of the NAMA-2 catalyst: full survey spectrum (a), and core level spectra of Ti 2p (b), C 1s (c), O 1s (d), F 1s (e), Mo 3d (f), S 2p (g), and Ni 2p (h).

The XPS technique was used to investigate the surface electronic states of the available elements in the NAMA-2 catalyst and their terminal groups. The XPS survey spectrum revealed Ni, Mo, S, Ti, C, O, and F elements (Fig. 4a) and so corresponding high-resolution XPS spectra were obtained for Ni 2p, Mo 3d, S 2p, Ti 2p, C 1s, O 1s, and F 1s (Fig. 4b). The photoemission of the Ti 2p region in the Ni-promoted MoS_2_/Ti_3_C_2_T_*x*_ (NAMA-2) catalyst was deconvoluted and the peaks were fitted, as shown in Fig. 4b. The Ti 2p spectrum showed three peaks corresponding to O or OH terminated groups placed at binding energies of 454.9, 456.0, and 457.6 eV, and corresponding to Ti–C, Ti^2+^–C and Ti^4+^–C, respectively.^38,41^

The C 1s spectrum of the NAMA-2 catalyst was deconvoluted to four peaks, with the major peak at +284.7 eV attributed to adventitious carbon (C–C) caused by material exposure to the atmosphere. The other peaks at 283.2, 285.8, and 287.4 eV corresponded to Ti–C bonds, surface C–O bonds, and C–F groups, respectively.^42,43^ The O 1s profile for the NAMA-2 catalyst was deconvoluted to four peaks located at 529.6, 531.2, 532.8, and 533.8 eV and assigned to TiO_2_, C–Ti–O_*x*_, Al_2_O_3_, and H_2_O(ads). The binding energies of some atoms and groups overlapped with many other peaks due to surface organic contamination. The peak value of C–Ti–O (531.2 eV) was close to that for O atoms near to vacancies in TiO_2_ (531.5 eV). The strongly adsorbed water molecules on the surface, and OH groups at bridging sites of titania appeared at the same binding energy of 533.8 eV. The F 1s spectrum was well fitted^43^ to four peaks, of which the main peak for C–Ti–F_*x*_ was located at 685.1 eV, and the peaks for TiO_2−*x*_F_*x*_, AlF_*x*,_ and Al(OF)_*x*_ appeared at 683.8, 687.0 and 689.1 eV, respectively.

The Mo 3d region displayed seven peaks, as shown in Fig. 4f. The two peaks for Mo–S bonds located at 228.9 and 231.9 eV corresponded to Mo^4+^ 3d_5/2_ and Mo^4+^ 3d_3/2_, respectively.^39,41^ Furthermore, a peak was observed at a lower binding energy (225.3 eV) which could be ascribed to S 2s of MoS_2_. The peaks of Mo–C bonds appeared at 227.7 and 231.8 eV, corresponding to Mo 3d_5/2_ and Mo 3d_3/2_, respectively. The higher oxidation state Mo^6+^ was also noticed and could be attributed to the partial oxidation of the Mo edges in MoS_2_ from the Mo^4+^ to Mo^6+^ state (MoO_3_ and MoO_4_^2−^) when exposed to the air. The peaks of Mo^6+^ located at 232.8 and 235.2 eV were ascribed to Mo^6+^ 3d_5/2_ and Mo^6+^ 3d_3/2._ The S 2p_3/2_ and S 2p_1/2_ peaks in the S 2p spectrum at 161.5 and 162.9 eV, respectively, corresponded to a doublet of S^2−^ species in MoS_2_ (Fig. 4g). The peak at 163.4 eV, ascribed to C–S–C bonds, was due to the strong intercalation of MoS_2_ into the Ti_3_C_2_T_*x*_ support. Another peak was observed at a high binding energy (169.4 eV), which coincided with the S–O bond, and might be due to the partial oxidation of the S edges in MoS_2_ by exposure to the atmosphere. The Ni 2p spectrum of the NAMA catalyst (Fig. 4h) showed two sets of doublets from Ni 2p_3/2_ (NiMoS phase) and Ni 2p_1/2_ at 855.4 and 873.2 eV, respectively, as well as satellite (Sat.) peaks at 860.7 and 880.0 eV, which were ascribed to the Ni^2+^ valence state.^43^ The other two peaks at 857.6 and 873.8 eV corresponded to Ni^3+^ (Ni_2_O_3_). In the ATM decomposition process, MoS_4_^2−^ converts to MoS_2_ and evolves H_2_S, which in turn causes slight sulfidation of NiO to NiS_*x*_, giving a peak located at 853.0 eV, and another small satellite peak at 864.0 eV. Also, the deconvolution of the Ni 2p spectrum indicated the presence of nickel oxide in the catalyst.

**Fig. 5:**
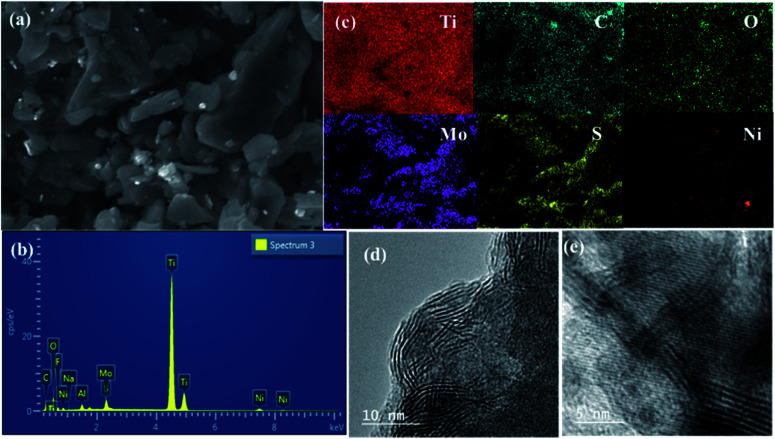
The surface morphology and elemental distribution of the NAMA-2 catalyst: SEM image (a), EDS spectrum (b), and elemental mapping (c). HRTEM images of AMA-2 (d) and NAMA-2 (e).

The SEM image of the NAMA-2 catalyst revealed the surface morphology and homogeneous intercalation of the promoter with the MoS_2_ nanosheets in the Ti_3_C_2_T_*x*_ support, giving an accordion-like structure (Fig. 5a). The EDS spectrum of NAMA-2 confirmed the presence of elements Ti, Mo, S, Ni, and C, and firmly indicated the existence of NiO, MoS_2,_ and Ti_3_C_2_T_*x*_ (Fig. 5b). The distribution of Ni, Mo, S, Ti, C, and O elements in NAMA-2 (NiMoS_2_/Ti_3_C_2_T_*x*_) was confirmed by elemental mapping analysis (Fig. 5c), which also confirmed the uniform distribution of promoter and MoS_2_ nanosheets on the surface and in between the layers of MXene. Further confirmation of the MoS_2_ nanosheets in the AMA-2 and NAMA-2 catalysts was given by the high-resolution transmission electron microscopy (HRTEM) images, as shown in Fig. 5d and e. The few monolayers of active MoS_2_ formed a hierarchical structure and the layer-to-layer spacing on the MXene was found from the TEM images;^42^ during the synthesis of the catalysts, 2D MoS_2_ formed as layer-structured crystallite stacks or slabs. The average number of layers per slab and slab length per stack were determined from the HRTEM morphology.^44,45^ The AMA-2 (MoS_2_/MXene) catalysts displayed a higher slab number of about 4.2 layers, and length of 6.4 nm compared to NAMA-2 (Ni–MoS_2_/MXene) which showed 3.4 layers and a slab length of 3.7 nm. This significant morphology difference between the NAMA-2 and AMA-2 catalysts might be due to the intercalation of layered MoS_2_ into MXene by the *in situ* thermal conversion process. Thus, the intercalation of 2D MoS_2_ stacks between the MXene slabs could be associated with the enhanced HDS activity of the NAMA catalysts. Moreover, the data from the Raman spectroscopy supported the formation of few-layered MoS_2_, and indicated about 2–3 layers.

**Fig. 6:**
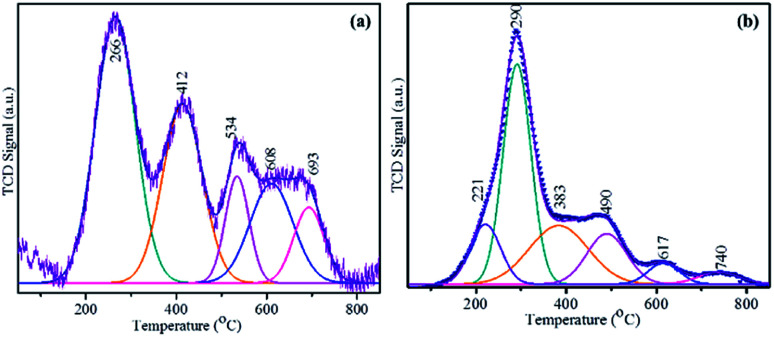
H_2_-TPR curves of the AMA-2 and NAMA-2 catalysts.

The H_2_-consumption of the prepared MoS_2_-based catalysts at a range of temperatures was obtained by temperature-programmed reduction (TPR) (Fig. 6). The heterostructured MoS_2_/Ti_3_C_2_T_*x*_ (AMA-2) or NiMoS_2_/Ti_3_C_2_T_*x*_ (NAMA-2) was placed in a quartz cell and pretreated at 150 °C in an Ar environment. A reducing gas containing 10% H_2_ in Ar was introduced into the sample quartz tube at a constant flow rate over the pretreated catalyst with a constant heating ramp of 10 °C min^−1^ up to 800 °C. The reduction of MoS_2_ on the Ti_3_C_2_T_*x*_ support was recorded based on H_2_ consumption signals by a thermal conductivity detector. The reduction peaks of labile sulfur moieties on the surface of the MoS_2_ supported catalysts (AMA and NAMA) were observed between around 200–800 °C. The AMA-2 signals were deconvoluted to five peaks located at 266, 412, 537, 608, and 693 °C, indicating sulfur dimers in the MoS_2_ layers. The edge sulfur atoms connected either with one or two Mo atoms, corresponding to hydrogen consumption signals at 266 and 412 °C, and another sulfur atom strongly bonded with Mo was evident in the range of 500–700 °C. The reduction of the NAMA-2 catalyst showed lower temperature peaks at 221, 290, 383, 490, and 617 °C, and Mo deeply intercalated in the support was reduced at 740 °C. The appearance of these reduction peaks at lower temperatures for the NAMA catalyst could be ascribed to the different reduction states of Mo due to the promoter Ni^2+^ placed next to Mo^4+^ edge ions and coordinated with S^2−^ ions, which reduced the strong interaction of Mo with the support;^46^ this was also confirmed by XPS data (Fig. 4). The H_2_-TPR profiles of AMA and NAMA in the low-temperature region between around 250–300 °C indicated reduction either by excess sulfur, or by sulfur weakly bonded to the support surface.

Furthermore, the weakly attached sulfur atoms easily consumed H_2_ on the surface of MoS_2_ to form H_2_S, in a continuous process. Hence, these processes generated sulfur vacancies in the transition sulfide, in turn, enhancing catalytic activity. Also, as supported by DFT theory,^47^ physisorbed H_2_ could split into separate atoms that adsorbed on adjacent sulfur dimers present in the catalyst, forming two adjacent S–Mo–SH surface groups. Then, an H atom was transferred from one S–Mo–SH to form SH–Mo–SH groups. The chemisorbed sulfur led to the desorption of H_2_S and the other sulfur atom associated with the same Mo edge. Thus, the NAMA-2 catalyst TPR curve indicated different reduction states: Mo^6+^ to Mo^4+^, Mo^4+^ to Mo^0^, and Ni^2+^ to Ni^0^.^48^

### Hydrodesulfurization activity on the MoS_2_/Ti_3_C_2_T_*x*_ and NiMoS_2_/Ti_3_C_2_T_*x*_ catalysts

The few-layered MoS_2_ decorated on Ti_3_C_2_T_*x*_ was formulated with or without promoter (Ni) by impregnation with aqueous ATM alone, or with ATM and then the nickel salt, and subsequent calcination in an inert medium. The Mo loading varied, with samples prepared at 6, 9, and 12 wt% on Ti_3_C_2_T_*x*_. These were denoted AMA-1, AMA-2, and AMA-3, respectively in the absence of promoter. The NiMoS_2_/Ti_3_C_2_T_*x*_ catalysts were intercalated by dispersion of promoter in ATM/Ti_3_C_2_T_*x*_ before calcination, with molar ratios of Ni/(Ni + Mo) of 0.39, 0.30, and 0.24 for the 6, 9, and 12 wt% Mo loadings, denoted NAMA-1, NAMA-2, and NAMA-3, respectively. The sulfur removal activity of each catalyst was investigated by using thiophene. Also, the above-mentioned catalysts were sulfided using H_2_S (to give S-AMA and S-NAMA), and their HDS activities were compared with those of the parent catalysts (AMA and NAMA). Typically, HDS activity was studied by passing saturated thiophene vapor over a catalyst loaded in a fixed-bed reactor, and the flow of thiophene concentration was controlled through a saturator system maintained at 5 °C. The amount of thiophene vapor from the saturator was calculated as 3.75 mol% using the Antoine eqn (1). The reaction was carried out at 350 °C, and the reactor outlet was connected to an online refinery gas analyzer for quantifying the desulfurized products and unreacted thiophene.^31^ The thiophene HDS reaction over AMA and NAMA catalysts showed the formation of significant amounts of C_4_ hydrocarbons (butane and butenes) and considerable amounts of C_1_–C_3_ hydrocarbons.

**Fig. 7:**
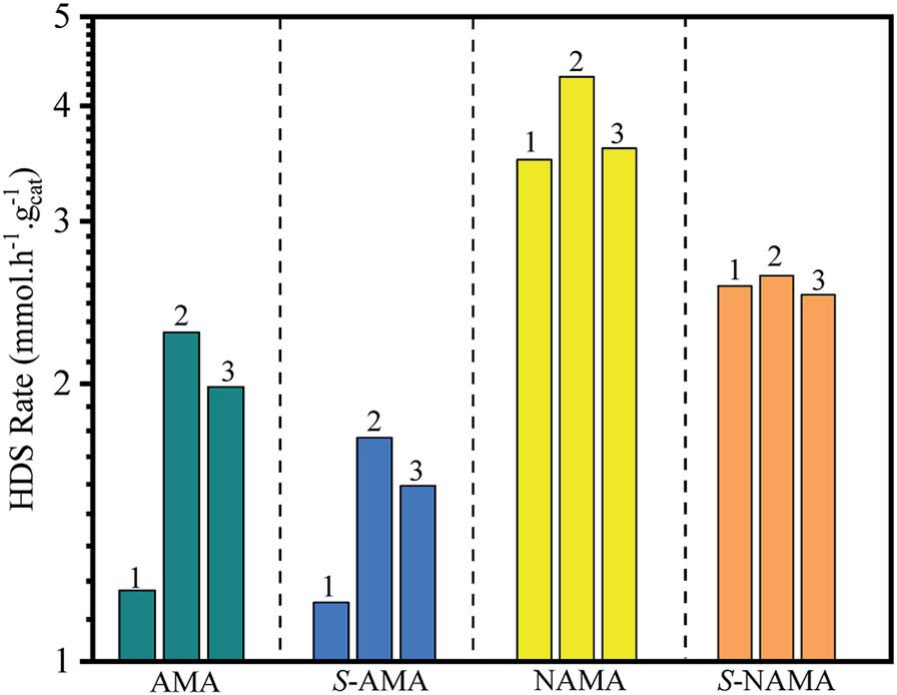
The comparison of the HDS activity of AMA and NAMA catalysts, and the conventional H_2_S-treated AMA and NAMA catalysts.

The HDS of thiophene was carried out in triplicate over the AMA and NAMA catalysts and the rate of thiophene conversion was determined by taking the average value for each catalyst. Fig. 7 shows the HDS rates of the AMA-1, AMA-2, and AMA-3 catalysts, which were 1.2, 2.27, and 1.9 mmol h^−1^ g^−1^, respectively, while the conversion rates of the Ni-based NAMA-1, NAMA-2, and NAMA-3 catalysts were 3.5, 4.3, and 3.6 mmol h^−1^ g^−1^, respectively. It has previously been reported that traditional Ni–MoS_2_/Al_2_O_3_ exhibited HDS activity of about 2.5 mmol h^−1^ g^−1^.^49^ Thus, in comparison with the reported alumina catalysts, Ni–MoS_2_ on Ti_3_C_2_T_*x*_ showed higher HDS rates, which might be due to the synergistic effect of the 2D MoS_2_ in 2D Ti_3_C_2_T_*x*_ with the Ni promoter. Fig. 7 reveals that the HDS rates of the AMA and NAMA catalysts increased with increasing Mo loading up to 9 wt%, but upon a further increase in Mo loading, the activity decreased, which might be due to the nature of MoS_2_ intercalated between the MXene layers. The 9 wt% Mo showed higher conversion rates compared to the other two concentrations, which might be due to the metal–support Ti_3_C_2_T_*x*_ interactions, which also play a substantial role in the conversion rates; even when the Mo concentration was increased from 6 to 12% wt, a maximum conversion rate cut-off was achieved at 9 wt% Mo.^50^ However, the NAMA catalysts showed higher conversion rates than the AMA catalysts due to the synergic effect between the Ni particles sited at the surface of the S-layers, and the layer edges of active MoS_2_ sites which dominated on the surface of C–Ti–O. Further, the Raman spectra results illustrate that the frequency difference between theE^1^_2g_ and A_1g_ modes in NAMA-3 was nearly 23 cm^−1^; the increase in the frequency difference could be attributed to the formation of close-packed multilayered MoS_2_. Thus, the formation of close-packed multilayered MoS_2_ with higher Mo loading might result in the lower catalytic activity of AMA-3, and NAMA-3.

In addition, the HDS of thiophene was investigated with H_2_S/H_2_-treated S-AMA and S-NAMA catalysts. The AMA and NAMA catalysts exhibited about 20% higher catalytic activities than the H_2_S/H_2_-treated S-AMA and S-NAMA catalysts. When compared with a similar study of H_2_S-treated Co–MoS_2_/alumina catalysts derived from ATM,^51^ the activity of NAMA-2 was about 7-fold higher than that of the H_2_S-treated Co–MoS_2_/alumina catalysts, due to the synergistic effect of Ni and MoS_2_ between the Ti_3_C_2_T_*x*_ slabs. Nevertheless, the decrease in HDS activity for the H_2_S/H_2_-treated S-AMA and S-NAMA catalysts might be due to inhibition of edge active MoS_2_.

**Fig. 8:**
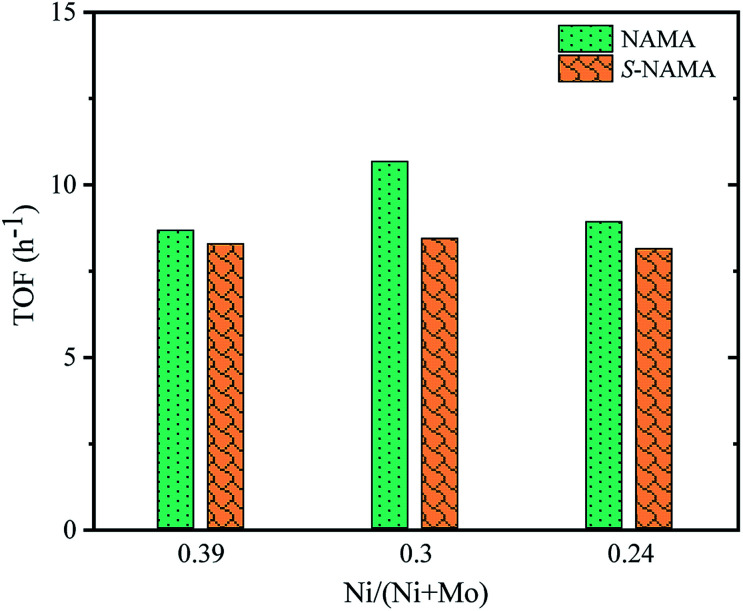
TOF on Ni–MoS_2_/MXene catalysts as a function of promoter concentration.

The turnover frequency (TOF) for the HDS of thiophene on Ni–MoS_2_/MXene was calculated from the ratio between the HDS activity of each NAMA catalyst (mol g^−1^ h^−1^) and the amount of Ni present in that NAMA catalyst (mol g^−1^).^52^Fig. 8 shows the TOF as a function of the Ni/(Ni + Mo) ratio for the NAMA and S-NAMA catalysts. The highest TOF value was achieved with a Ni/(Ni + Mo) ratio of 0.30 (NAMA-2) which exceeded the values for NAMA-3 (low Ni content) and NAMA-1 (high Ni content). This confirmed that NAMA-2 was more intrinsically active compared to NAMA-1 and NAMA-3 due to the sintering of the active phase.

A number of products were obtained through the HDS of thiophene that were formed through various intermediates by hydrogenation of unsaturated carbon bonds and direct S removal from C–S bonds.^47^ The MoS_2_-based catalysts undergo parallel HDS reactions: the hydrogenation (C=C bond) involves the π-electrons of the S-ring compound adsorbed on the MoS_2_ layer, and direct S-removal (C–S bond) involves the exposed Mo ion and S vacancy site of chemisorbed S-atoms of thiophene *via* hydrogen transfer followed by sulfur elimination. These pathways involve different intermediates (dihydrothiophene, tetrahydrothiophene, and butadiene) and generate different products such as butenes (1-butene, *cis*-2-butene, *trans*-2-butene), butanes (*n*-butane, i-butane), and C_1_–C_3_ hydrocarbons (methane, ethane, ethene, propane, propene).^50^ The activities of the AMA and NAMA catalysts depended on the weakly bonded sulfur at the surface of the few-layered MoS_2_ for the generation of H_2_S, which created sulfur vacancies that reduced the energy barrier and favored the hydrogenation reaction, enhancing the catalytic conversion rates.

**Fig. 9:**
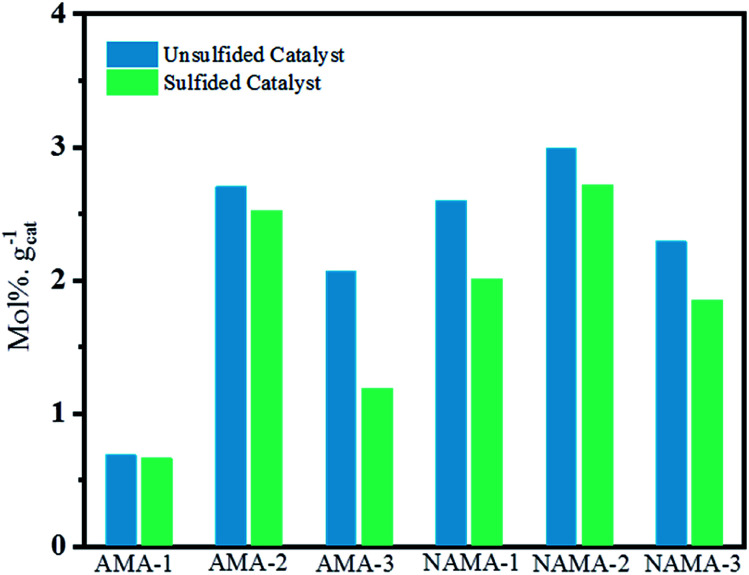
Effect of sulfidation on few-layered MoS_2_/MXene catalysts for overall thiophene conversion.

Fig. 9 shows the average thiophene conversion by the synthesized (AMA and NAMA) and sulfided (S-AMA and S-NAMA) catalysts in terms of mol% per gram of catalyst at a particular time (5 h). The synthesized few-layered MoS_2_/Ti_3_C_2_T_*x*_ catalysts revealed higher conversion than the H_2_S-sulfided catalysts because of the saturation of the active sites with surface carbon. The AMA catalysts displayed an appreciable thiophene conversion due to the presence of corner MoS_2_ that helped to cleave C–S bonds, and edge MoS_2_ that enabled hydrogenation of thiophene.^7,53^ However, the Ni-promoted NAMA catalysts were more active for the dissociation of hydrogen molecules and their association with the evenly dispersed MoS_2_ on the support which strengthened the MoS_2_ hydrogen capacity. Furthermore, MoS_2_ peaks were not noticed in the XRD patterns of the NAMA catalysts, which suggests that the nanocrystalline MoS_2_ and Ni were highly dispersed to form Ni–Mo–S structures. In other words, the thermal conversion of intercalated ATM and promoter precursor on Ti_3_C_2_T_*x*_ in an inert medium favored the formation of an active Ni–Mo–S phase, and in turn, this decreased the formation of NiS_*x*_ and NiO_*x*_, as confirmed by the XPS spectra. Thus, it is clear that the formation of the active Ni–Mo–S phase in the NAMA catalysts favored the high conversion of thiophene.

**Fig. 10:**
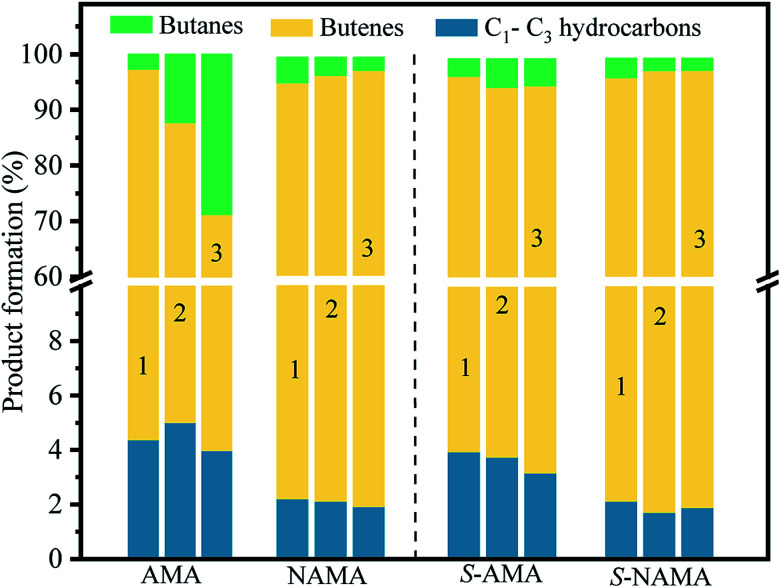
The distribution of HDS products on the parent AMA and NAMA catalysts, and the sulfided S-AMA, and S-NAMA catalysts.

Fig. 10 shows the product distributions obtained on the AMA and NAMA catalysts and the corresponding sulfided catalysts (S-AMA and S-NAMA). The results reveal a declining trend in the formation of butenes and increasing proportion of butanes upon increasing Mo loading on the AMA catalysts, which favors the hydrogenation of thiophene; the conversion of low carbon products (C_1_–C_3_) remained the same at about 3–5% after completion of the HDS reaction. However, this change was not observed on the S-AMA catalysts due to the hidden edge MoS_2_ sites and so the distribution of butenes, butanes, and C_1_–C_3_ hydrocarbons corresponded to about ∼92%, ∼4%, and ∼4%, respectively. On the other hand, the NAMA and S-NAMA catalysts favored butenes (∼95% formation), while the formation of butanes and C_1_–C_3_ hydrocarbons slightly diminished over S-NAMA compared to those on NAMA catalysts. The sulfidation process might convert the active Ni promoter to inactive NiS_*x*_ on the surface of the S-NAMA catalysts, which could decrease the hydrogenation of thiophene, resulting in the lower C_1_–C_3_ and butane contents.^6,7^ Overall, it was observed that the NAMA catalysts gave about 98% of C_4_ (butenes and butanes) and about 2% of C_1_–C_3_ hydrocarbons. Thus, the aqueous ATM and 2D MXene (titanium carbide) offer a new opportunity for the formulation of a new generation of catalysts. Moreover, using ATM as the Mo precursor provides a better *in situ* approach than a conventional hazardous H_2_S sulfidation process.

## Conclusions

Few-layered MoS_2_ with and without Ni-promoted Ti_3_C_2_T_*x*_ (AMA and NAMA) catalysts were successfully prepared from the precursor ATM ((NH_4_)_2_MoS_4_) which was obtained in aqueous form by purging H_2_S through ammonium heptamolybdate. The AMA and NAMA catalysts were sulfided using H_2_S, to produce S-AMA and S-NAMA catalysts. The absence of MoS_2_ peaks in the XRD patterns of the AMA catalysts indicated the formation of highly dispersed nanocrystalline MoS_2_ on Ti_3_C_2_T_*x*._ The intercalation of MoS_2_ and promoter between the layers of Ti_3_C_2_T_*x*_ and the number of MoS_2_ layers formed were confirmed from the textural properties of the catalysts and the active A^1^_g_ and E^1^_2g_ Raman spectroscopy modes. HRTEM images and scanning electron microscopy coupled with energy dispersive X-ray spectroscopy (SEM-EDS) mapping revealed the catalyst surface morphology and elemental distribution. The electronic states of the elements on the surface of Ti_3_C_2_T_*x*_ that catalyzed the thiophene HDS were established by XPS analysis. The kinetic parameters were established from the conversion rates, and the formation of C_1_–C_3_, and C_4_ hydrocarbon products was quantified with GC. The overall reaction rate for HDS was higher for the NAMA than for the AMA catalysts due to the enhancement in rate provided by the promoter Ni. Furthermore, the prepared AMA and NAMA catalysts showed higher HDS rates than the corresponding sulfided catalysts because the H_2_S-sulfidation led to the poisoning of active MoS_2_ sites. The product distribution showed higher C_4_ (butane and butene) formation than C_1_–C_3_ product formation on the layered MoS_2_-MXene catalysts. The NAMA-2 catalyst with the 0.30 molar ratio of Ni/(Ni + Mo) exhibited the highest HDS rate and showed significant selectivity for *n*-butane and butenes. XRD and XPS data indicated that the active Ni–Mo–S structure formed on Ti_3_C_2_T_*x*_ for thiophene HDS through the hydrogenation and hydrogenolysis of C–S bonds. In summary, the epitaxial synthesis of HDS catalysts from ATM and 2D MXene provides an alternative route to conventional metal oxide sulfidation by H_2_S. Thus, the Ni-promoted few-layered MoS_2_/Ti_3_C_2_T_*x*_ catalyst is a potential candidate for achieving an enhanced HDS rate in the clean fuel process.

## Conflicts of interest

There are no conflicts to declare.

## Supplementary Material

RA-010-D0RA01158D-s001
