# Supplementary material for: Epitaxial synthesis of Ni–MoS2/Ti3C2Tx MXene heterostructures for hydrodesulfurization
Source: RSC Adv. 2020 Mar 26;10(21):12308–17. doi: 10.1039/d0ra01158d (PMC9050720; doi:10.1039/d0ra01158d)
Supplement: RA-010-D0RA01158D-s001 [file RA-010-D0RA01158D-s001.pdf]

Electronic Supplementary Material (ESI) for RSC Advances.
This journal is © The Royal Society of Chemistry 2020

Supplementary Information

Epitaxial Synthesis of Ni-MoS_2/$Ti_3C_2T_x$ MXene Heterostructures for Hydrodesulfurization

Mari Vinoba*, R. Navvamani, Hanadi Al-Sheeha

Petroleum Research Center, Kuwait Institute for Scientific Research, Kuwait.

Materials

Titanium aluminum carbide (Ti_3AlC_2, Purity > 98 wt%) was provided by Hangzhou Dayangchem Co., Ltd., P. R. China. Analytical grade of ammonium heptamolybdate ($(NH_4)_6Mo_7O_{24}\cdot4H_2O$), nickel nitrate ($Ni(NO_3)_2.4H_2O$), hydrofluoric acid (HF), thiophene and ammonium hydroxide solution were obtained from Sigma Aldrich and used without further purification. Milli-Q water was used to prepare solutions. The gas cylinders of 10 % H_2S/Ar, 10 % H_2S/H_2, N_2, and H_2 were purchased from Refrigeration & Oxygen Co. Ltd. Kuwait.

Characterization

The X-ray diffraction (XRD) patterns of Ti_3AlC_2, $Ti_3C_2T_x$, and prepared catalysts were collected from PANalytical PW3040 (Cu-Kα radiation, λ=0. 1542 nm) with X'pert PRO software to verify the formation of MXene from MAX phase. N_2 adsorption-desorption isotherms were measured at 77 K on Micromeritics ASAP 2020 for estimation of surface area, pore-volume, and pore diameter of catalysts. The number of MoS_2 layers formed in the catalysts was calculated from Raman spectroscopy on Senterra, Bruker. The catalysts were analyzed by HRTEM using a JEOL-2000EX operated at 120 kV. The surface morphology and distribution of active metals of the target catalysts

were obtained by scanning electron microscopy (SEM) with energy dispersive spectroscopy (EDS) using a JEOL-JSM-IT300 with Oxford-EDS. To understand the surface-active metal binding energies of catalyst by X-ray photoelectron spectroscopy (XPS), Thermo Scientific K-Alpha spectrometer. H_2 temperature-programmed reduction (H_2-TPR) of the catalysts was conducted with AMI-300S, Altamira Inc, USA.

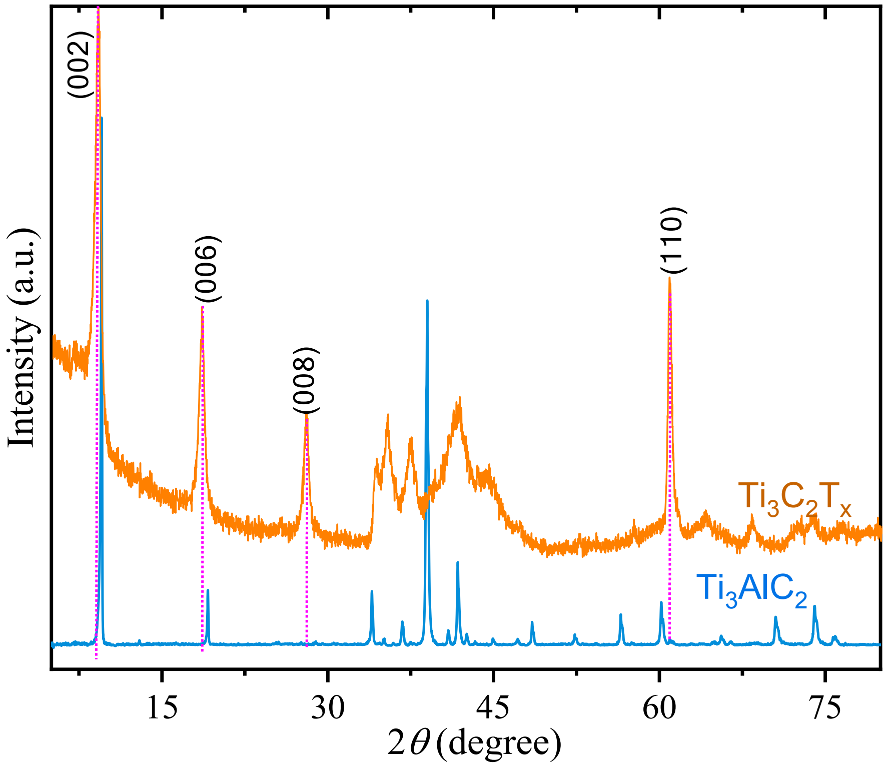

Fig. S1 XRD diffraction patterns of MAX phase (Ti_3AlC_2) and $Ti_3C_2T_x$ MXene.

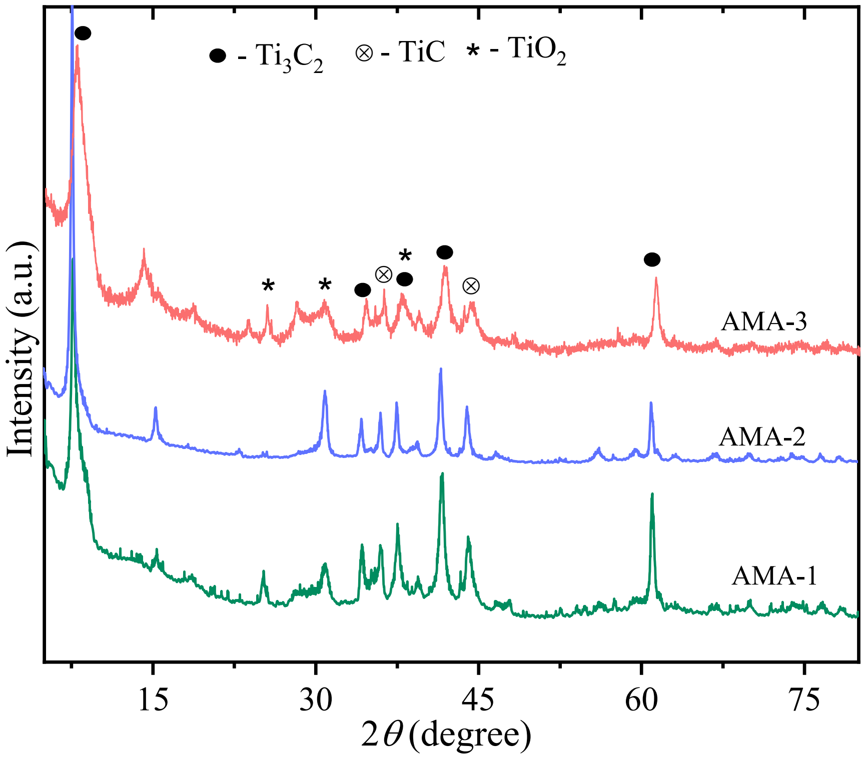

Fig. S2 XRD diffraction patterns of $MoS_2/Ti_3C_2T_x$ (AMA) at different Mo loading.

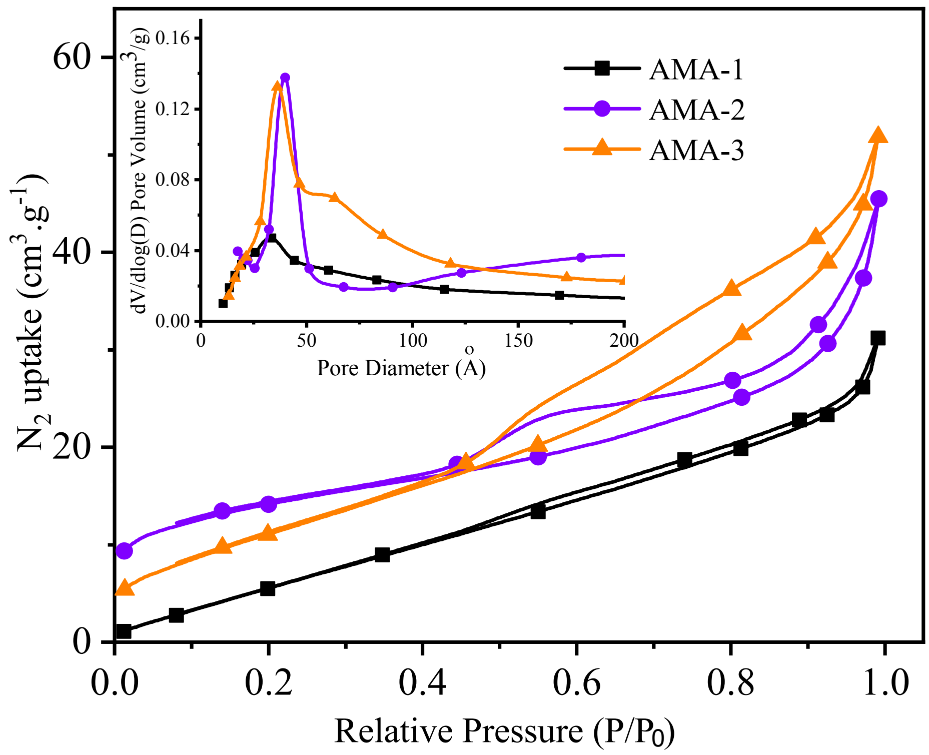

Fig. S3 The textural properties of AMA catalysts, and ascribed to their hysteresis loop and pore size.
